# GLP-1 receptor agonist as a modulator of innate immunity

**DOI:** 10.3389/fimmu.2022.997578

**Published:** 2022-12-08

**Authors:** Jun Chen, Aihua Mei, Yingying Wei, Chunlei Li, Hang Qian, Xinwen Min, Handong Yang, Lingli Dong, Xiaoquan Rao, Jixin Zhong

**Affiliations:** ^1^ Sinopharm Dongfeng General Hospital, Hubei University of Medicine, Hubei Key Laboratory of Wudang Local Chinese Medicine Research (Hubei University of Medicine), Shiyan, China; ^2^ Department of Rheumatology and Immunology, Tongji Hospital, Tongji Medical College of Huazhong University of Science and Technology, Wuhan, Hubei, China; ^3^ Department of Cardiology, Tongji Hospital, Huazhong University of Science and Technology, Wuhan, China

**Keywords:** glucagon-like peptide-1, liraglutide, inflammatory diseases, immunomodulation, signal pathways

## Abstract

Glucagon-like peptide-1 (GLP-1) is a 30-amino acid hormone secreted by L cells in the distal ileum, colon, and pancreatic α cells, which participates in blood sugar regulation by promoting insulin release, reducing glucagon levels, delaying gastric emptying, increasing satiety, and reducing appetite. GLP-1 specifically binds to the glucagon-like peptide-1 receptor (GLP-1R) in the body, directly stimulating the secretion of insulin by pancreatic β-cells, promoting proliferation and differentiation, and inhibiting cell apoptosis, thereby exerting a glycemic lowering effect. The glycemic regulating effect of GLP-1 and its analogues has been well studied in human and murine models in the circumstance of many diseases. Recent studies found that GLP-1 is able to modulate innate immune response in a number of inflammatory diseases. In the present review, we summarize the research progression of GLP-1 and its analogues in immunomodulation and related signal pathways.

## 1 Introduction

Glucagon-like peptide-1 (GLP-1) is an endogenous incretin secreted by small intestinal L cells, consisting of two bioactive isoforms: GLP-1 (7-37) and GLP-1 (7-36). GLP-1 is rapidly degraded by an enzyme named dipeptidyl peptidase-IV (DPP-4), converting to bioinactive products GLP-1 (9-36) and GLP-1 (9-37) (1–3), which have a very low affinity for GLP-1 receptor (GLP-1R) and have no insulin secretion effect. Endogenous GLP-1 has a short plasma half-life of about 2-3 min (4–6).

GLP-1 exerts its function by binding to GLP-1R and is involved in the development and progression of many diseases (7, 8). GLP-1R is a family of transmembrane G protein-coupled receptor B that was originally found in islet β cells but is widely expressed in extra pancreatic tissues, including the lungs, kidneys, central nervous system, enteric nervous systems, lymphocytes, blood vessels, and kidneys (9–11). Binding of GLP-1 with its receptor enhances glucose-stimulated insulin secretion and reduces glucagon. GLP-1 also delays gastric emptying, increases satiety, and reduces appetite. Therefore, it lowers blood glucose through multiple mechanisms (12, 13). The interaction between GLP-1 and GLP-1R exerts a variety of physiological functions, including promoting insulin synthesis and secretion, inhibiting islet cell production and releasing glucagon, reducing hepatic glycogen output, acting on the central nervous system, increasing satiety, and reducing food intake, by activating different downstream signaling molecules (14–16).

GLP-1R agonists (GLP-1RAs) are a group of GLP-1 analogues that are resistant to DPP4-mediated degradation, working through activating GLP-1R and its downstream signaling (3, 17, 18). GLP-1RA is widely used to treat type 2 diabetes by enhancing insulin production, and they also have the added benefit of suppressing appetite and losing weight (19, 20). GLP-1RAs are also involved in the nervous, cardiovascular, and endocrine systems (21–23).

The expression of GLP-1R remains controversial due to the lack of specific antibody against GLP-1R, with most of the literatures using qPCR to detect mRNA levels (24, 25). There were also a few studies detected the expression of GLP-1R at the protein level using a lineage-tracking animal model (26, 27). Although Glp1r mRNA transcripts have been detected in murine lymphoid tissue, little is known about the role of GLP-1 in the immune system (28). Here, we will discuss in-depth the actions of GLP-1 and its analogues in immunomodulation and related signal pathways in the setting of a number of diseases.

## 2 GLP-1 in regulating immune system

### 2.1 The role of GLP-1 in innate immune cells

Recent studies have demonstrated that GLP-1 and its analogues exert regulatory functions in innate immune cells, especially macrophages. It has been shown that GLP-1 analogue exenatide could activate the human monocyte-derived macrophage towards M2 phenotype (29). Lixisenatide, another GLP-1 analogue, has also been reported to decrease atheroma plaque size and instability in animal models, by reprogramming macrophages towards M2 phenotype (10 μg/kg/day) (30). Besides, Exendin-4 could promote the polarization of bone marrow-derived macrophages into M2 subtype (4.2 µg/kg/day) (31). These studies indicate that the GLP-1 and its analogues may act directly on macrophages and polarize the macrophages to M2 phenotype. Some other studies revealed that GLP-1 and its analogues may also indirectly promote M2 polarization by suppressing M1 and enhancing regulatory T cells (Tregs). DPP4 inhibitor alogliptin, which preserves GLP-1, was able to reduce visceral adipose tissue macrophage content in LDLR^-/-^ mice with a concomitant upregulation of M2 marker CD163 (32). Another DPP4 inhibitor Des-fluoro-sitagliptin was also reported to decrease the accumulation of M1 macrophages in Gck^+/-^mice, a β-cell–specific glucokinase haploinsufficient (Gck+/−) diabetic model (33). Liraglutide, a widely used GLP-1RA in clinic, ameliorated the macrophage accumulation in periodontitis, by decreasing M1 macrophages but not M2 macrophages (30 μg/kg/day) (34). GLP-1 could directly reduce the M1 polarization by modulating the JNK/STAT3 activation in a murine monocyte/macrophage cell line RAW264.7 (35). In addition, GLP-1 administration also enhances the Treg function (36), which may promote M2 polarization (37). In a recent clinical study, we reported that the expression of GLP-1R on macrophages, especially M2 marcrophages, was significantly reduced in patients with coronary heart disease (CHD), suggesting a potential role of macrophage-derived GLP-1R signaling in cardiovascular disease (38). In addition to macrophages, human neutrophils and eosinophils have also been shown to express GLP-1R and GLP-1 signaling significantly decreased the expression of eosinophil-surface activation markers along with a decrease in the production of IL-4, IL-8 and IL-13 (39). Taken together, GLP-1 and its analogues play a crucial role in innate immune cells, especially in macrophage.

### 2.2 The role of GLP-1 in innate-like T lymphocytes

Invariant Natural Killer T (iNKT) cells are a subpopulation of T lymphocytes that bridge the innate and adaptive immune systems (40). A. E. Hogan, et al (10). reported that GLP-1R on the surface of iNKT could trigger downstream signal transduction cascades. GLP-1 inhibits the secretion of IFN-γ and IL-4 by iNKT cell in a dose-dependent manner. Dermal γδ T cells are another innate-like T lymphocyte subset that evoke innate and adaptive immune responses. They play a very important role in psoriasis patients with type 2 diabetes, and the administration of a GLP-1 analogue improved the psoriasis severity by decreasing the dermal γδ T-cell number and IL-17 expression (41).

### 2.3 The role of GLP-1 in intestinal epithelial lymphocytes

As a innate like lymphocytes, intestinal epithelial lymphocytes (IELs) patrol the lamina propria and play a critical role in host-bacteria interaction, wound healing, mucosal growth and regeneration, and inflammation (42). Tαβ and Tγδ IELs represent the two major subsets of intestinal IELs, and GLP-1R was expressed in both of these subsets at a higher level than cells isolated from the spleen, lymph nodes, and bone marrow (43). In addition, the activation of GLP-1R by exendin-4 increased cAMP accumulation and reduced cytokine production in IELs. CD4+ CD25+ Tregs are a subpopulation of T cells that are effective in reducing inflammation (44, 45). A significantly lower percentage of peripheral Tregs was detected in naive male GLP-1R^-/-^ mice, while the numbers of CD4+ and CD8+ T cells and B cells in the spleen and lymph nodes had no differences (36). In addition, the activation of GLP-1R can increase Treg frequency and function, as demonstrated by flow cytometry and inhibition assays in diabetic NOD mice, with increased IL-10 expression and enhanced cellular inhibitory function (46). Th1/Th17 cell is a T cell subset which co-expresses IFN-γ and IL-17A (47), driven by IL-12 or IL-23 (47, 48). The development of tissue-infiltrating interferon (IFN)-γ/interleukin (IL)-17A doubly-secreting encephalitogenic CD4-positive T cell subset in the CNS was notably inhibited by dulaglutide (49). CD4-positive T lymphocytes was suppressed by liraglutide, accompanied by improved hepatosteatosis and metabolic function in female mice (200 μg/kg twice daily) (50). In a T-cell-dependent glomerulonephritis model, T-cell proliferation and nephritis was inhibited by liraglutide 200 μg/kg once daily (51). In conclusion, GLP-1 and its analogues have an important immunomodulatory effect in innate like lymphocytes.

## 3 The role of GLP-1/GLP-R axis in related signal pathways

GLP-1 is known to increase insulin secretion by β cells under hyperglycemic conditions. Although the GLP-1R agonists are used to treat type 2 diabetes in clinic (52, 53), there are direct evidences about the therapeutic actions of GLP-1-based therapies in different healthy conditions in humans, including adipogenesis, osteogenesis, and nociception, with many signaling pathways are involved (54).

### 3.1 PKA/STAT3 pathway

GLP-1 and its analogues exert their function such as M2 polarization by inducing the activation of the activator of transcription 3 (STAT3) (29). Following the treatment with GLP-1, the phosphorylation of JNK and its signal transduction through the cyclic adenosine monophosphate/protein kinase A (PKA) signaling pathway was attenuated, while the phosphorylation of STAT3 was increased, which further induce the polarization of macrophage towards M2 (35). An ovariectomized and a macrophage-depleted model were employed to investigate the effects of Exendin-4 on macrophages and bone formation. The results showed that Exendin-4 also promoted bone marrow-derived macrophage polarization to M2 phenotye and TGF-β1 secretion *via* PKA-STAT3 signaling (31). Non-alcoholic fatty liver disease (NAFLD) induced by a high-fat diet was used to investigated the Kupffer cells M2 polarization in the liver, which shows that liraglutide can reverse the negative effects of nonalcoholic fatty liver disease by modulating Kupffer cell M2 polarization *via* the cAMP-PKA-STAT3 signaling pathway (55).

### 3.2 MAPK and NF-κB pathway

Mitogen-activated protein kinase (MAPK) pathway was also involved in the signaling of GLP-1R (56–59). Male ob/ob mice were subcutaneously injected with liraglutide daily for 4 weeks, fatty acid synthase (FASN) was down-regulated through the MAPK/ERK and PKA signaling pathways (11). In an *in vitro* study with peripheral blood mononuclear cells, exendin-4 suppressed inflammatory responses and reduced oxidative stress, which was rmediated by suppressing MAPK signaling pathway (60). The protective effects of GLP-1 on IL-6 production and high glucose-induced endothelial progenitor cells (EPCs) dysfunction also mediated by the MAPK signaling pathway (61, 62). In a partial hepatectomy-induced behavior study using male Sprague-Dawley rats, surgical trauma induced an exacerbated spatial learning and memory impairment, while exendin-4 treatment suppressed the activation of nuclear factor kappa-B (NF-κB) and IL-1β, and thus ameliorated hepatectomy-induced behavioral deficits and inflmmation (63). Similarily, GLP-1R-mediated suppression of NF-κB p65 was also able to modulate neuropathic pain-induced neuroinflammation and improve recognition memory dysfunction (64).

### 3.3 PI3K/Akt pathway

GLP-1RAs can also function through the Phosphoinositide 3-kinases (PI3K)/AKT pathway (65–67). Microvascular endothelial cells (CMECs) were isolated from neonatal Sprague–Dawley (SD) rat hearts by the enzyme dissociation to induce hypoxia/reoxygenation (H/R) injury, and GLP-1 analogue liraglutide protected cardial microvascular endothelium from H/R injury by activating PI3K/Akt/survivin pathways (68).Liraglutide-induced PI3K activation was also able to enhance keratinocyte migration and promote wound healing in mice (9). MC3T3-E1 cells were incubated with liraglutide, which directly acts on osteoblasts and activates PI3K/AKT signaling, promoting bone formation (69). However, there were also a few reports suggesting that PI3K/Akt pathway was inhibited by GLP-1 and its analogues. Pancreatic cancer cell lines and mouse xenograft models of human pancreatic cancer were used to evaluate the effects of the GLP-1R agonist liraglutide *in vitro* and vivo. The results demonstrated that GLP-1R activation with liraglutide dose-dependently suppressed Akt activation and tumourigenicity/metastasis in human pancreatic cancer cells both *in vitro* and *in vivo* (70).

### 3.4 Other pathways

In addition to the above mentioned signal pathways, GLP-1/GLP-R axis also also activates a number of other important signal pathways. Chang’s findings suggest that EX-4 inhibited LPS-induced iNOS expression at protein level, but not at transcriptional level, of iNOS gene *via* a cAMP/PKA dependent mechanism (71). Liraglutide can reduce oxidative stress and fatty degeneration in oxLDL-treated Raw264.7 cells, accompanied by an alteration of AMPK/SREBP1 pathway (72). In consistency, AMPK activation induced by GLP-1 impaired inflammatory signals in keratinocytes and restrained macrophage migration (73).

## 4 The role of GLP-1/GLP-R in related disease

### 4.1 Diabetes

Diabetes mellitus is characterized by increased inflammation, reflecting innate immune control disorders, and studies have shown a local intestinal intraepithelial lymphocyte (IEL)-GLP-1 receptor (GLP-1R) signaling network that controls the mucosal immune response (43). GLP-1 agonists, a novel class of anti-diabetic drugs, are an integral part of the management of patients with type 2 diabetes (74–76). GLP-1 agonists bind to GLP-1 receptors on pancreatic β cells, which directly stimulates pancreatic β cells to secrete insulin. They can also promote the proliferation of pancreatic β cells, increase the number of β cells, inhibit apoptosis, and promote insulin synthesis, thus resulting in hypoglycemic effects (77, 78). GLP-1RA has also been reported to promote motor activity and energy expenditure, thus activating the metabolism of brown fat in rodents (20). Weight-loss effect of non-insulin glucose lowering drugs in patients with type 2 diabetes is also a hot spot of current research. A systemic analysis shows that GLP1-RA and Tirzepatide are most effective in inducing weight loss in patients with type 2 diabetes among a variety of anti-diabetic drugs (79). Studies have suggested that in addition to diabetes, GLP-1R agonists may also have a beneficial effect on many other diseases such as cardiovascular disease, central nervous disorder, and tumors (21–23).

### 4.2 Cardiovascular disease

The latest research has found that GLP-1R is widely expressed in the cardiovascular system and is involved in intracellular metabolism and signal transduction. These metabolites are biologically active, can reduce intravascular oxidative stress, inhibit hepatocyte gluconeogenesis and oxidative stress, increase cardiomyocyte activity, promote vasodilation, protect the cardiovascular system, improve cardiac function, thereby directly or indirectly protecting the cardiovascular system (80–82). For example, a number of large-scale clinical trials have demonstrated that GLP-1RA could reduce the risk of cardiovascular events, which has been well reviewed in elsewhere (83, 84) and we will not further discuss here.

### 4.3 Nervous system disorder

Recently, a protective effect of the GLP-1/GLP-1R axis on ischemic brain injury has been emphasized. The activation of GLP-1R can reduce the size of cerebral infarction by enhancing cell survival signaling pathways, reducing ischemia-reperfusion injury, promoting brain repair, and inhibiting inflammatory response and oxidative stress (85–88). As we mentioned before, GLP-1R agonism is able to ameliorate neuroinflammation and behavioral deficits induced by hepatectomy or neuropathic pain (63, 64). In addition, activation of GLP-1R in the brain is albe to reduce appetite, lowering the risks for other diseases such as metabolic and cardiovascular disorders (88).

### 4.4 Tumors

The role of GLP-1RA in tumor remains controversial. Due to the promotive effect of GLP-1R agonism on β-cells proliferation and survival, concerns of tumorigenesis, especially pancreatic cancers, have been raised about incretin-based therapies (89). By analyzing the reported adverse events in the US Food and Drug Administration’s database, Elashoff reported that treatment with DPP4 inhibitor sitagliptin or GLP-1R agonist exenatide increased the risk of pancreatitis and pancreatic cancer as compared with other therapies. However, other types of cancer occurred at a similar level between patients who took sitagliptin and those with other therapies (90). Animal study has also demonstrated that exenatide can promote pancreatic duct hyperplasia, a well-established risk factors for pancreatic cancer (91). However, a meta-analysis including 37 eligible randomized controlled trials failed to identify an association between GLP-1RAs and increased risk of overall cancer. There was even a lower risk of overall cancer in patients treated with albiglutide in the subgroup analysis (92). Another meta-analysis on pancreatic cancer specifically also showed that GLP-1RAs were not associated with increased risk for pancreatic cancer as compared with other treatments (93). Interestingly, GLP-1R has been utilized to mediate tumor specificity for insulinoma that highly expresses GLP-1R during radiotherapy. A single injection of an Ahx-DTPA-(111)In)NH(2) In-labeled GLP-1R agonist markedly reduced tumor volume in a dose-dependent manner in a mouse model of insulinoma (94). Mechanistical studies suggest that GLP-1RAs may reduce tumor growth in prostate (95) and breast cancer (96, 97). Taken together, the exact role of GLP-1R in tumorigenesis remains to be illucidated.

### 4.5 Asthma

Asthma is a very common chronic lung disease characterized by chronic persistent airway inflammation and airway remodeling, resulting in incompletely reversible airway obstruction, especially in advanced stages (98). Many researchs showed that GLP-1R signaling inhibits the innate immune response in animal models of asthma, by the activation of PKA/NF-κB signaling pathway (99) and the decreased eosinophil production of IL-4, IL-8 and IL-13 (39). GLP-1RA treatment may be a new pharmacologic adjunctive treatment strategy for obese patients with asthma (100, 101).

## 5 Discussion

**Figure 1:**
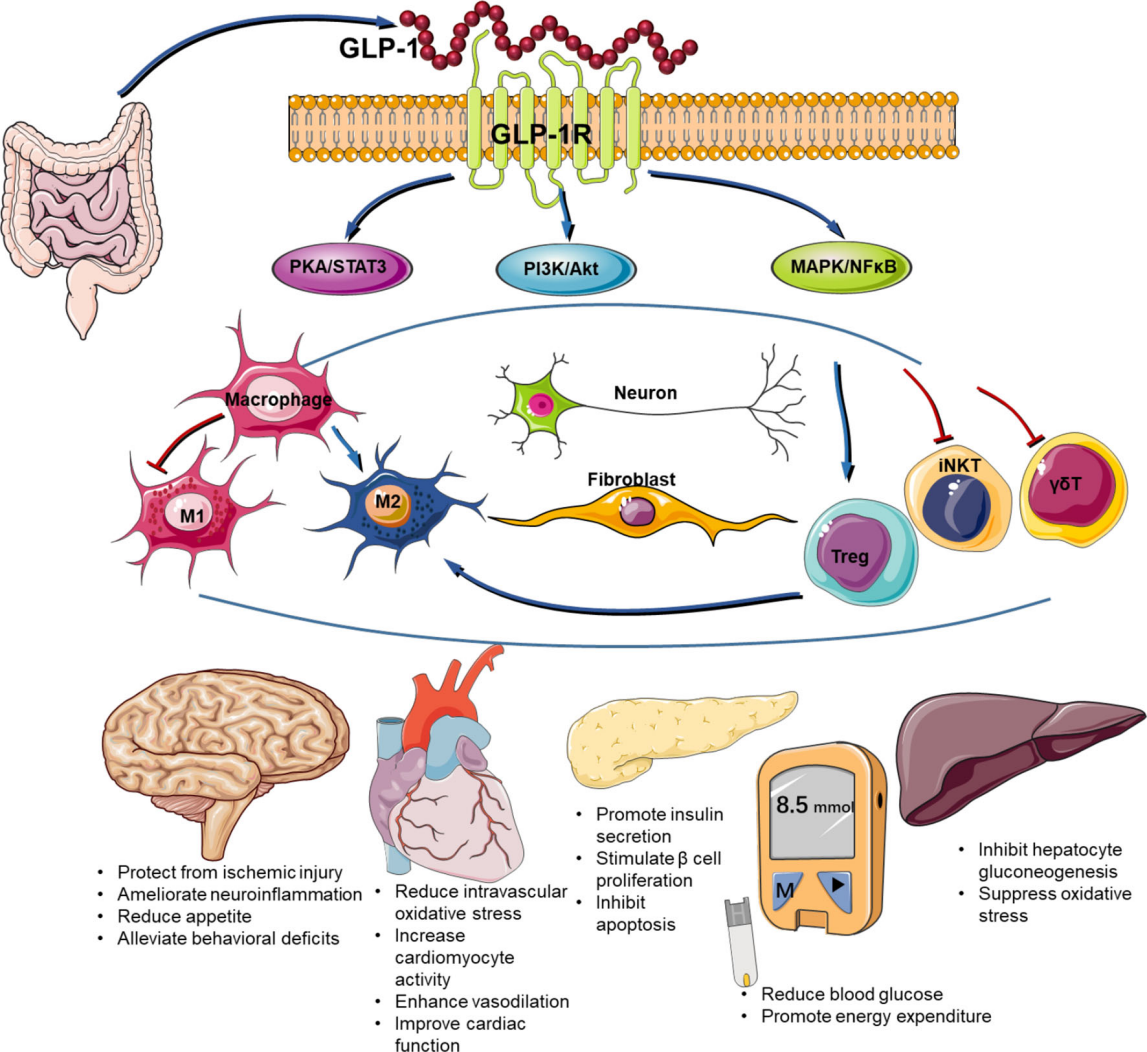
Immune regulatory function of GLP-1/GLP-1R axis.

The safety and efficacy of GLP-1RAs in the treatment of type 2 diabetes have been well demonstrated. Recent investigations suggest that it also participates in a number of other diseases by regulating immune response (**Figure 1**). Both the innate and innate-like cells express GLP-1R. Engagement of GLP-1R and its ligands activates multiple signaling pathways including PKA/STAT, PI3K/Akt, MAPK, and NFκB. Given the importance of inflammation in disease progression, GLP-1RAs have been shown to possess beneficial effects on many other diseases in addition to diabetes. For example, the improvements in cardiovascular outcome have been evidenced in a number of large-scale randomized controlled clinical trials on cardiovascular disease (102). The obvious improvement of skin lesions in patients with psoriasis type 2 diabetes mellitus after liraglutide treatment may be related to inhibition of the expression of inflammatory factors such as IL-23, IL-17, and TNF-α (103). Despite of recent advances in our understanding of the immune regulatory role of GLP-1/GLP-1R, there are many challenges to overcome in this area. First, the expression of GLP-1R remains controversial in many types of cells and tissues due to the lack of specific antibody against GLP-1R. Second, the exact molecular signaling for GLP-1R activation remains elusive. Last, to what extend immune system is involved in the beneficial effects of GLP-1RAs on cardiometabolic and other diseases is not well understood. Therefore, further studies are required to delineate the detailed mechanisms by which GLP-1RAs regulate immune function and chronic inflammatory diseases.

## Author contributions

JC, AM, YW, CL, HQ, XM, HY, and LD searched the data, JC, XR, and JZ wrote the main manuscript text. All authors reviewed and revised the manuscript. All authors contributed to the article and approved the submitted version.
